# Insights into Nucleation and Growth of Colloidal Quaternary Nanocrystals by Multimodal X-ray Analysis

**DOI:** 10.1021/acsnano.0c08617

**Published:** 2021-03-26

**Authors:** Justus Just, Claudia Coughlan, Shalini Singh, Huan Ren, Oliver Müller, Pascal Becker, Thomas Unold, Kevin M. Ryan

**Affiliations:** † MAX IV Laboratory, Lund University, Fotongatan 2, 22484 Lund, Sweden; ‡ Department of Chemical Sciences and Bernal Institute, University of Limerick, V94T9PX Limerick, Ireland; § Stanford Synchrotron Radiation Lightsource, SLAC National Acceleration Laboratory, Menlo Park, California 94025, United States; ∥ Department of Structure and Dynamics of Energy Materials, Helmholtz-Zentrum Berlin für Materialien und Energie GmbH, 14109 Berlin, Germany

**Keywords:** colloidal nanocrystals, wurtzite, CZTS, nucleation and growth, quick-EXAFS, in situ EXAFS/XANES/SAXS

## Abstract

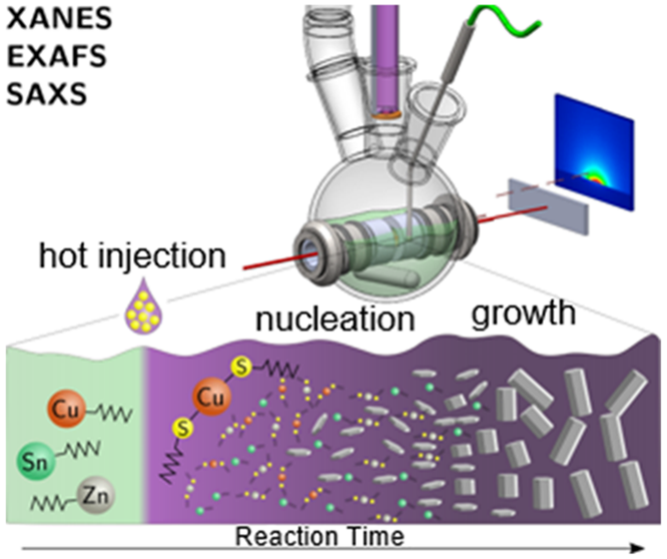

Copper chalcogenide nanocrystals find applications in photovoltaic inks, bio labels, and thermoelectric materials. We reveal insights in the nucleation and growth during synthesis of anisotropic Cu_2_ZnSnS_4_ nanocrystals by simultaneously performing in situ X-ray absorption spectroscopy (XAS) and small-angle X-ray scattering (SAXS). Real-time XAFS reveals that upon thiol injection into the reaction flask, a key copper thiolate intermediate species is formed within fractions of seconds, which decomposes further within a narrow temperature and time window to form copper sulfide nanocrystals. These nanocrystals convert into Cu_2_ZnSnS_4_ nanorods by sequentially incorporating Sn and Zn. Real-time SAXS and ex situ TEM of aliquots corroborate these findings. Our work demonstrates how combined in situ X-ray absorption and small-angle X-ray scattering enables the understanding of mechanistic pathways in colloidal nanocrystal formation.

## Introduction

Colloidal nanocrystal (NC) syntheses have progressed to the point where excellent control of size and shape allows for a multitude of applications that utilize carefully tuned electronic and optical properties such as displays, biotags, and photovoltaics.^1^ While much work has been devoted to developing adaptable solution approaches to control crystal phase and morphology, the underlying mechanistic processes leading to nanocrystal nucleation and subsequent growth processes are still poorly understood.^2−9^ Real-time insights into nucleation and growth are highly desirable to understand in greater depth the solution processes and intermediates that facilitate the formation of these inorganic particles with such a high degree of control.

Here we look at the compound multielement copper chalcogenide Cu_2_ZnSnS_4_ (CZTS) NCs, which are related to the binary copper chalcogenide system by the additional incorporation of Sn and Zn metal cations.^10^ This allows for compositionally tunable optical band gaps with high optical absorption coefficients and good photostability.^11−15^ In addition, the natural abundance and relatively low toxicity of these elements makes them suitable for a more sustainable chemistry.^11,16^ In this colloidal NC system, the presence of three different metal cations in combination with a chalcogen anion requires judicious selection of precursors, solvents, and ligands in combination with optimized reaction protocols to deliver nearly monodisperse particles.^17,18^ Furthermore, we have shown that these particles can be stabilized in wurtzite form that is not stable in the bulk, allowing anisotropic control for the formation of nanorods with defined aspect ratio.^19−23^

Recent investigations on CZTS NCs have focused on investigating their evolution pathway through the use of in situ X-ray diffraction (XRD),^24^ grazing- incidence small-angle and wide-angle X-ray scattering (GISAXS/GIWAXS),^25^ surface-enhanced Raman scattering (SERS),^26^ as well as transmission electron microscopy (TEM) and energy-dispersive X-ray (EDX) analysis of aliquots extracted from the reaction flask at desired intervals.^27−30^ A disadvantage of these techniques is that they are not sensitive to the temporal evolution of the atomic species and the state of nascent nuclei that typically dominate the reaction within tens of seconds of the combination of precursors by hot injection.^6,31^

In situ X-ray absorption spectroscopy (XAS) is an element-specific and time-resolved technique^32−34^ with two regimes, namely, X-ray absorption near-edge structure (XANES) and extended X-ray absorption fine structure (EXAFS). These contain information about the elements local coordination and chemical state.^33^ The absorption edge of the XANES region is highly sensitive to the local chemical environment, such as oxidation states and unoccupied electronic states.^35,36^ The EXAFS region starts after the XANES region (a few tenths of eV) and extends to several hundred eV.^32,33,36^ This region is sensitive to the radial distribution of electron density around the absorbing atom and allows quantitative determination of bond length and coordination number.^33,35^ In situ EXAFS measurements on CdSe NCs at the Se K-edge have been reported and reveal that CdSe nucleation completes within several seconds upon injection of the anionic precursor.^37,38^ EXAFS measurements have also been acquired post synthesis on CdTe,^39,40^ CZTS,^41^ and CZTSSe^42^ NCs.

**Figure 1:**
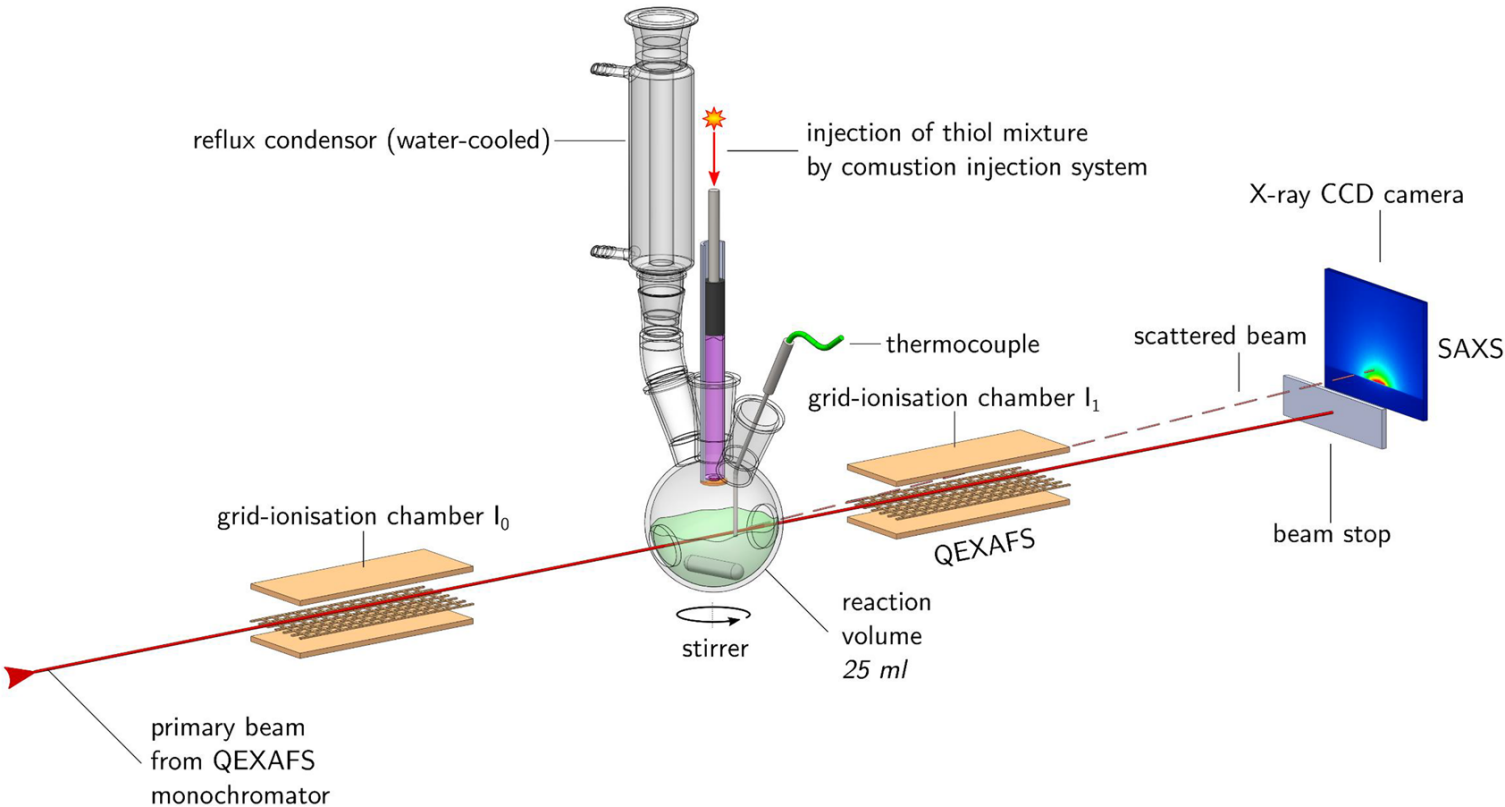
Schematic drawing of the experimental setup used at the SuperXAS beamline of the SwissLightSource. The primary beam is monochromatic and oscillating in energy with a frequency of 40 Hz covering the near edge and extended X-ray absorption fine structure of the Cu and Zn K-edges.

Herein, we show the capability of quick-EXAFS (QEXAFS) in situ measurements in combination with simultaneous small-angle X-ray scattering SAXS (Figure 1) to unravel the key nucleation and growth stages in CZTS NCs with respect to the different precursors. The results give important insights into the temporal occurrence of key reaction intermediates during the nucleation and growth processes and allows for insights into the growth mechanism where rod growth is concurrent with a compositional change from a binary to quaternary compound copper chalcogenide.

## Results and Discussion

**Figure 2:**
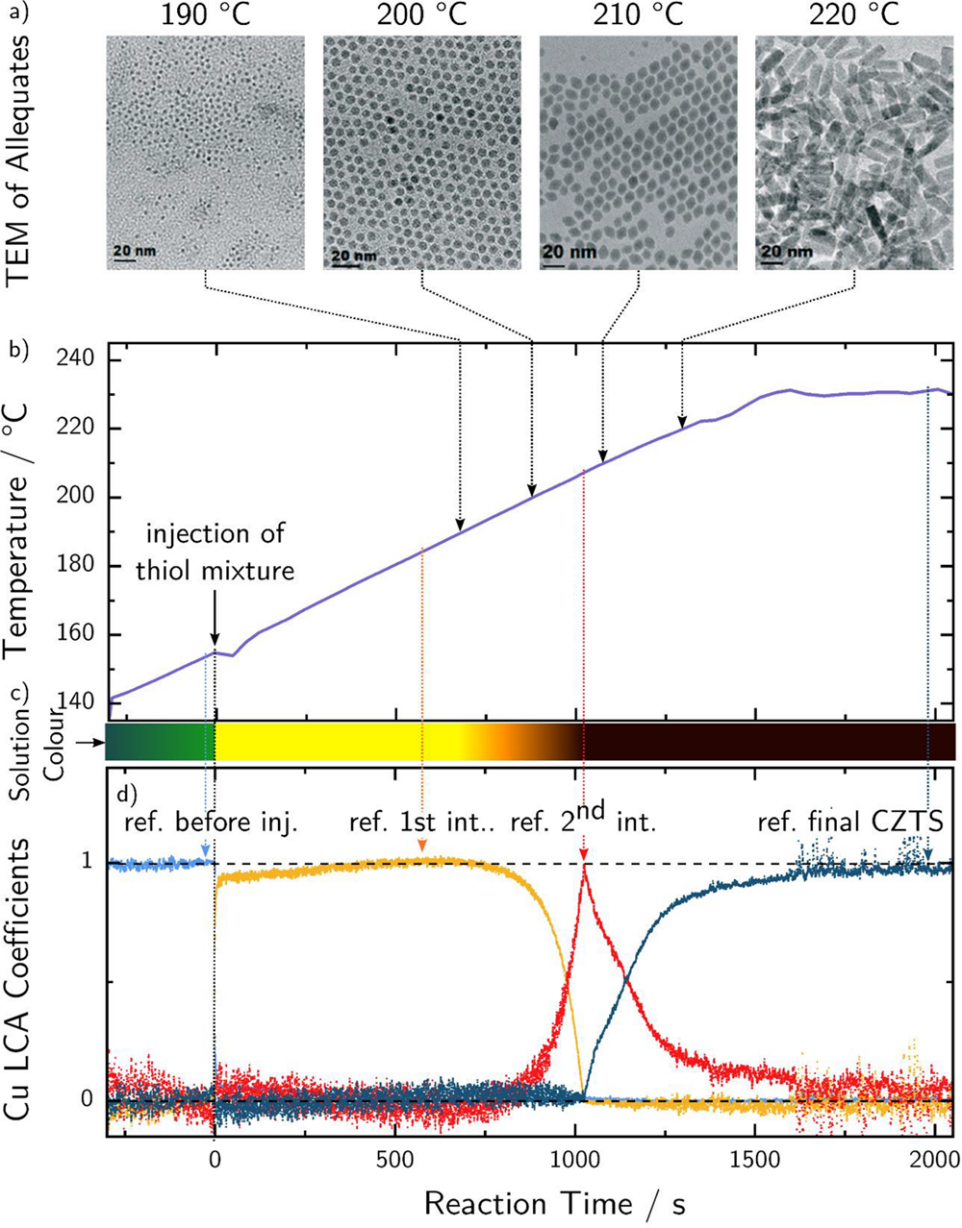
Overview of the reaction kinetics of CZTS NC nucleation and growth upon thiol mixture injection: (a) TEM images of the aliquots at different temperatures. (b) Temperature profile of the reaction as measured by a liquid-surrounded thermocouple. (c) Schematic evolution of the solution color. (d) Reaction kinetics as extracted from in situ XAS at the K-edge of Cu by linear combination analysis using internal reference spectra from the indicated points of the reaction.

All recorded X-ray absorption spectra were fitted with a linear combination of reference spectra taken from the reaction itself (full details and assumptions are discussed in Section S1, SI). By means of this internal linear combination analysis (LCA), four reference points are identified on the Cu K-edge during the reaction: (1) before injection, where metal salts are in solution; (2) after the injection of the anion precursor (1st intermediate); (3) a second reaction intermediate; and (4) the final state, consisting of CZTS nanorods. The evolution of the LCA coefficients of those reference points over the whole reaction is shown in Figure 2, together with the timeline of external reaction parameters such as the temperature of the solution and its color. Aliquots are taken from a similar reaction at different points in time. TEM images of those are shown in Figure 2a as a comparison.

It can clearly be seen from the LCA timelines of the Cu K-edge that copper forms a first reaction intermediate immediately after injection of the anionic precursor at 155 °C, where the solution color changes accordingly from green to yellow. This first intermediate is stable over a wide range of temperatures up to approximately 190 °C. At higher temperatures, the second intermediate state is formed under consumption of the first, reaching a maximum in concentration around 1000 s after injection, which corresponds to a temperature of 207 °C. During this transition, the solution color changes from yellow over orange to dark brown. Finally, the final state (CZTS) starts to form and reaches a plateau in concentration at around 230 °C.

**Figure 3:**
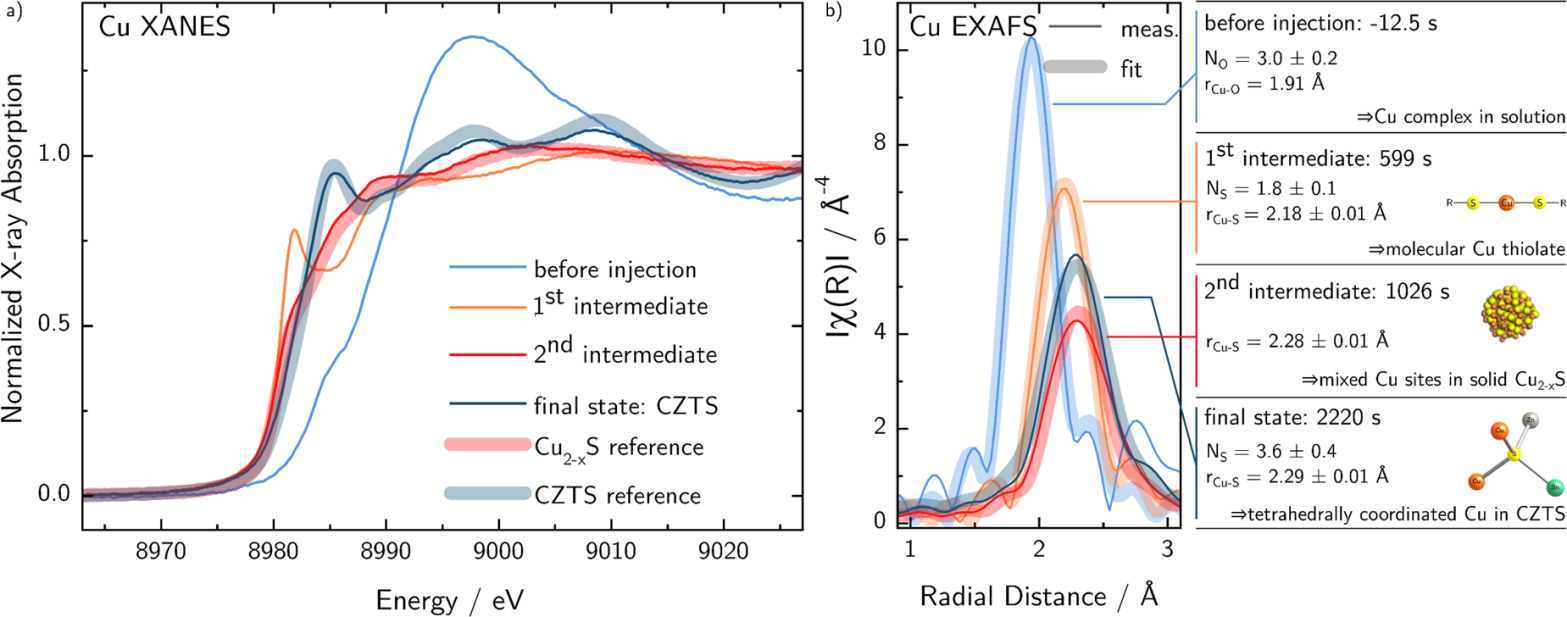
Extracted Cu K-edge X-ray absorption spectra measured in situ at different times of the reaction as indicated in Figure 2. (a) Edge-step normalized X-ray absorption near edge structure (XANES) together with powder reference spectra of polycrystalline copper sulfide and stoichiometric kesterite CZTS. (b) Phase-corrected Fourier transform of the EXAFS oscillations measured at different times of the reaction together with a fitted ab initio model to extract coordination numbers and bond lengths, see section S1, SI.

To further identify the different stages of the reaction or the different species where copper is involved, the XANES and EXAFS parts of the Cu K-edge absorption spectra are analyzed individually in detail. Figure 3a shows the extracted internal reference spectra corresponding to the reference points of the LCA shown in Figure 2, together with external reference spectra for stoichiometric CZTS powder and Cu_2–*x*_S NCs. CZTS reference spectra are measured on stoichiometric Kesterite powder reference samples as described elsewhere.^43^ Note that even though wurtzite CZTS is expected here, the spectrum is compared to that of Kesterite CZTS as the wurtzite structure is unstable for bulk materials such as powder references. However, since the local surrounding of copper atoms is very similar (tetrahedral coordination with sulfur), the Cu-XANES of Wurtzite and Kesterite CZTS are expected to be very similar or even undistinguishable. The copper sulfide reference spectrum stems from the final state of a similar reaction as the herein investigated, where the anionic precursor was injected into a cationic solution of Cu only.

Taking the comparison of XANES spectra as shown in Figure 3a, it becomes clear that all identified reference points exhibit significantly distinct XANES spectra, justifying the LCA analysis for extraction of the reaction kinetics. The final state of this reaction can be identified as CZTS, as supported further by TEM EDX analysis additionally, see Section S2, SI. The XANES of the second intermediate state agrees well with that of the copper sulfide reference NCs. As shown in Figure S2.1, SI, it is also significantly different from that of copper tin sulfide. Therefore, the second intermediate can be identified as solid state (NCs) containing copper and sulfur, only.

Additional information about the nature of the reference points of the reaction can be concluded from the EXAFS region of the spectra. In order to extract meaningful EXAFS spectra with a high signal-to-noise ratio the raw data was treated applying a Savitzki–Golay filter^44^ (SG) with a width of 500 spectra and a fourth-order polynomial. These settings ensure the best quality of the spectra while still not smearing out the features of the temporarily appearing second intermediate. SG filtered timelines of Figure 2d as well as the extracted k^3^-weighted spectra can be found in Figures S1.2 and S3.1 in the SI, respectively, while the Fourier transform of the EXAFS oscillations is shown in Figure 3b.

First-shell EXAFS oscillations are analyzed by fitting to an ab initio model generated by FEFF with help of the IFEFFIT software package.^45^ By this analysis, the elemental nature of the next nearest neighbor to Cu can be unambiguously determined. Additionally, the coordination number is estimated, and the radial distance between Cu as a central atom and its next nearest neighbor is determined. Details of the EXAFS fitting procedure can be found in Section S3, SI.

Analysis of the spectrum taken before injection of the anionic precursor shows that Cu is coordinated by three oxygen atoms (average) with a bond distance of 1.9 Å, typical for a Cu–O bond. The XAS edge position of this spectrum is located at about 8985 eV, revealing that the oxidation state of Cu is 2+.^46^ The edge position of all other reference spectra is around 8982 eV, resulting in an oxidation state of 1+ for copper after injection of the anionic precursor until the final CZTS.

The first intermediate state after injection of the anionic precursor shows copper bound to approximately two sulfur atoms with a radial distance of 2.18 Å. This low coordination number points out that at this state after injection Copper is still found in a molecular state and not yet in a nucleated solid species. However, because of the averaging nature of XAS, it cannot be excluded that a small percentage of copper atoms have created nuclei. Instead a stable molecular species, which can be identified as Cu thiolate, forms and prevents the instant formation of NCs. This might be an important prerequisite for the size- and shape-controlled formation of multielement NCs.

The second intermediate state, which was previously identified as copper sulfide shows copper in a sulfur co-ordination with a bond distance of 2.28 Å. This is in very good agreement with the bond length distribution found in Djurleite or low-chalcocite and therefore gives further evidence that the second intermediate can be identified as solid copper sulfide NCs.^16,47^ The fit result, however, has to be interpreted carefully: The expected bond distance distribution in copper sulfide cannot correctly be treated by a single shell ab initio model. Considering shells with a significantly wider distribution of the radial distance, as expected for Djurleite or low-chalcocite copper sulfide, would result in a damping of the magnitude of the EXAFS oscillations due to interference. This damping is reflected in the as-determined EXAFS path degeneracy (too low) taking only one shell into account.

The final-state EXAFS oscillations show an expected coordination number around four with a bond distance of 2.29 Å. As expected, this can be assigned to tetrahedrally coordinated copper in Wurtzite CZTS, where the averaged sulfur cation (Cu, Zn, Sn) bond-length is 2.36 Å.^48^

**Figure 4:**
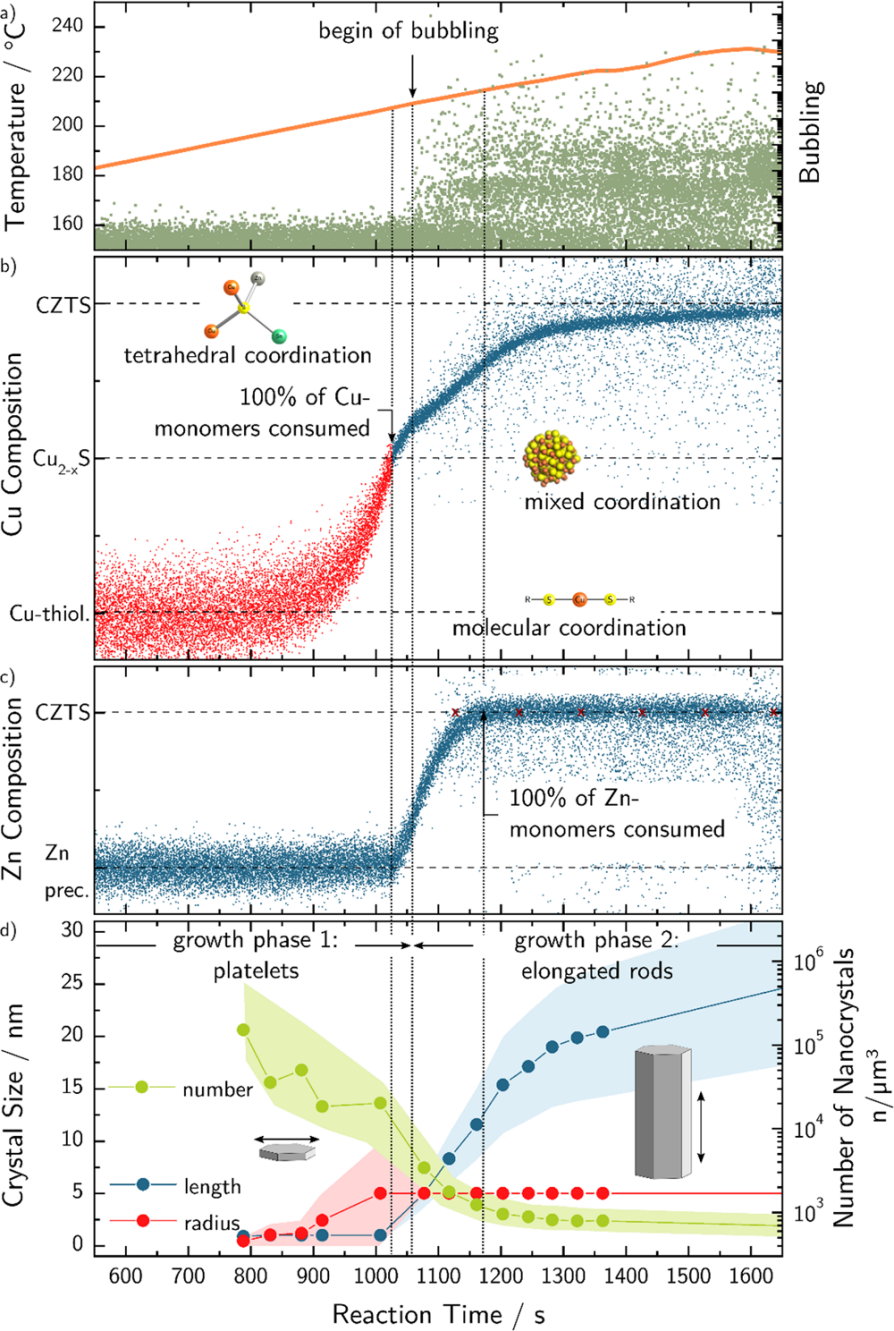
Transition timelines of the part of the reaction relevant for nanorod growth. Each data point represents the evaluation result of one individually measured spectrum/pattern: (a) Temperature of the reaction together with a measure for gas formation (bubbling) in the reaction flask which was extracted from discontinuities of the X-ray absorption baseline. (b, c) Quantitative measures of the binding states of Cu and Zn atoms, respectively, as determined by linear combination analysis of the XANES spectra. (d) Particle growth parameters as determined by fitting the SAXS patterns with the above-described model of partly agglomerated cylindrical crystals.

X-ray absorption spectra of the Zn K-edge have been acquired simultaneously and are treated the same way as described above for the Cu K-edge spectra. Zn-edge specific information is given in the Sections S1 and S3 (SI) for XANES and EXAFS analysis, respectively. Edge-step normalized near X-ray absorption spectra of the Zn K-edge have been acquired simultaneously and are treated the same way as described above for the Cu K-edge spectra. Zn-edge specific information is given in the Sections S1 and S3 (SI) for XANES and EXAFS analysis, respectively. Edge-step normalized near edge spectra as well as the phase-corrected Fourier transform of the EXAFS oscillations are shown in Figure S3.2, SI. Comparing the XANES of the final CZTS nanorods with the XANES of ex situ measured CZTS powder reference, it becomes evident that in the final state, 100% of the Zn-precursor material has been converted to solid state. To differentiate between CZTS and ZnS, the EXAFS oscillations of the Zn edge have to be analyzed. Figure S3.4 and S3.5 in SI show the extracted EXAFS at different points of the reaction, as indicated in Figure 4c, together with a spectrum of a ZnS equilibrated powder reference. The difference in the EXAFS of ZnS compared with that of CZTS is originating from the type of backscattering atom in the first metal shell, which is Zn in the case of ZnS and Cu/Sn in case of CZTS. Because of the interfering EXAFS oscillations in the case of a Cu/Sn mixture (see Figure S3.6, SI), the features of the first metal shell are substantially damped in the case of CZTS. It therefore can be concluded that no significant amounts of solid crystalline ZnS are present during the reaction. Disordered or amorphous ZnS particles can however not be excluded. Within the sensitivity of the XANES analysis, which can be estimated to about (3% of the total Zn), no Zn monomer is detected in the solution either. In contrast to the case of copper, the EXFAS oscillations of Zn before and after the injection are nearly identical, which provides evidence that the local coordination of Zn is unchanged. The spectra are fitted with ab initio models of the first coordination shell. Before and after injection, the fit shows a good agreement to the data with Zn only coordinated with (about four) oxygen atoms, which can be identified as the precursor complex. As expected, the final-state spectrum can be well explained by a tetrahedral sulfur coordination with a bond distance of 2.36 ± 0.06 Å. From here, it can therefore be concluded that the Zn remains in its original precursor state upon injection of the sulfur precursor and is then directly incorporated into CZTS nanorods during further reaction without the formation of persistent Zn intermediates. However, it cannot be excluded that an intermediate is formed and consumed at the same time, such that no significant amount is present at a time. As the Tin K-edge is at a significantly different energy (29200 eV), Sn spectra were not investigated in this work. It is therefore unclear whether the Sn precursor is changed upon injection or not.

### Reaction Kinetics

In the following, the kinetics of the reaction will be discussed in detail, considering the above-described analysis of the different intermediate stages. An overview of the reaction timeline for nanorod growth with all extracted information from in situ measurements is shown in Figure 4. Three critical points in the growth time have been designated (showed by dashed lines) as the important growth events.

While conducting the NC synthesis as described in the experimental section, it becomes apparent that the solution starts to bubble heavily above a certain threshold temperature. This can be attributed to the evaporation of the volatile byproducts formed during the coordination of thiol with metal precursors. In the initial stages of the reaction, metal thiolate formation occurs involving metal ions coordinating to a deprotonated thiol and the consequent release of short-chain carboxylic acids.^27,49^ The excessive effervescing of the reaction solution can be attributed to this, which is evident from the fluctuation of the effective density of the solution. In Figure 4a, the significant leaps in the edge step of the absorption edge during the course of the reaction is plotted together with the temperature of the reaction flask. It can be seen that the bubbling begins when the solution temperature exceeds 209 ± 1 °C.

Panels b and c of Figure 4 show the transition of the copper and zinc during the reaction, respectively. The data here directly corresponds to the LCA coefficient of the respective reference spectrum. Note that the value of the LCA coefficient of one phase is directly proportional to the number of Cu/Zn atoms bound in that specific phase. Therefore, panels b and c of Figure 4 represent the transformation of initial precursors to the final NC during the course of the reaction.

As can be seen in Figure 4b,c, copper thiolate which was formed upon injection, starts to decompose above 190 °C, while the zinc precursor remains stable. At 1021 s after the injection (∼207 °C), the copper thiolate is completely consumed (critical point **1**) to form copper sulfide solid state. Beyond this point, two changes occur simultaneously: (1) Cu_2–*x*_S NCs start to convert to CZTS nanorods; (2) the zinc precursor starts to decompose and releases zinc which is then incorporated into the growing particles to form CZTS. At critical point **2** (1052 s, ∼209 °C), the changes involve: (1) Vigorous bubbling starts to appear, (2) the consumption rate of Cu_2–*x*_S NCs changes to a slower rate indicating a change in crystal conversion mechanism, (3) about 20% of the Cu and Zn is found in the final CZTS phase. However, in the case of Zn incorporation, the Zn monomer consumption rate does not change at the second critical point. A possible explanation relates to the thiol acting as a surfactant while the surfaces of the NCs are preferentially copper terminated. It would follow that the Cu edge is more sensitive to changes on the crystal surface (e.g., growth on consumption of quasi 2D particles), while the transition observed at the Zn edge is dominated by the bulk. Hence, the slow continuing transition at the Cu edge is postulated to be due to the change of the surface of the NC due to growth.

To gain more insight into the growth of the nanorods, the simultaneously in situ recorded data of small angle scattering is analyzed. It has to be noted that the experiment was set up to achieve high data quality and time resolution for the X-ray absorption spectroscopy measurements, compromising on the data quality of the scattering patterns. Individual scattering patterns are shown in Figure S4.1 in the Supporting Information, together with details of data reduction and analysis. Significant agglomeration (large particle sizes) was observed in the scattering patterns leading to an overlapping Porod background to the single-particle scattering patterns. Considering these limitations, the interpretation of the results must be very careful and can only be considered as indicative rather than providing evidence for specific interpretations. However, fitting the individual SAXS patterns with the model of a cylinder together with a Porod-like back-ground still reveals valuable information about the growth mechanisms.

The growth model for the fit is chosen in agreement with the TEM images of the reaction aliquots: In a first regime, for times below about 1075 s after the injection, the cylinder z-dimension is fixed to 1 nm, while the radius is varied. The volume fraction for the fit is constrained to the volume fraction calculated based on the XANES measurements at the Cu k-edge. After this point in time when the reaction mixture starts bubbling, the radius is fixed in the fit, while the length is varied. The determined radius at this point is ∼5 nm, agreeing well with the radius determined from TEM pictures for the final CZTS rods.

The correlation between cation incorporation and morphological progression is analyzed in Figure 4d. Because of the integration time of the 2D detector, the time resolution is much lower for the SAXS patterns compared to the XAFS measurements. Nonetheless, it can be clearly observed in Figure 4d that the number of NCs reduce with an oscillating reduction rate before the complete consumption of Cu thiolate at critical point **1**. After this point, the reduction rate of the number of NCs is more steady between critical point **1** and **2**. The *z*-axis radius increases before the Cu thiolate consumption while the length remains unchanged. After this point, the radius stays steady, while the NC length increases significantly. Considering the TEM images of the NCs taken from aliquots at different temperatures, which are shown in Figure 2, it is evident that the nanorod growth in the direction of the *z*-axis occurs in the temperature range between 210 ± 5 °C and 220 ± 5 °C. In this range, already 100% of the copper thiolate has transformed into Cu_2–*x*_S; however, Zn monomers are only consumed partially. Considering these findings, the growth along *z*-axis can be attributed to the Zn incorporation and Cu_2–*x*_S consumption, while the cross-sectional diameter does not change significantly. Furthermore, the nanorods elongates faster during the fast Cu__2–*x*__S NCs consumption process from 1021 s (∼207 °C) to 1052 s (∼209 °C).

### Growth Model

These observations collectively provide a comprehensive picture of sequence of growth events occurring during the formation of CZTS nanorods: 1. Injection of thiol leads to the immediate formation of copper thiolate, which decomposes further to generate quasi 2D Cu_2–*x*_S NCs; 2. After acquiring the optimum radius of the final nanorods, these Cu_2–*x*_S NCs start elongating along the *z*-axis by incorporation of other cations. Interestingly, during this process, not all Cu__2–*x*__S nuclei develop into CZTS nanorods. In fact, a significant amount of Cu__2–*x*__S nuclei is consumed to sustain the incorporation of other cations in Cu__2–*x*__S NCs and growth of nanorods in later stages. Hence, up on the complete Cu thiolate consumption (critical point **1**), the source for NC elongation is mainly digestive growth of Cu__2–*x*__S (between critical point **1** and **2**). Once the critical point **2** is passed, NC elongation is continued with incorporation of other metal ions (Zn^2+^ and Sn^4+^) in the growing NCs. The heavy effervesce in the reaction flask at this stage also strongly indicates that the metal precursors are decomposing releasing volatile gases along with the metal ions in the solution for the incorporation in the growing crystals. After the full incorporation of Zn^2+^ (beyond critical point **3**), one sees that the nanorods continue growing along *z*-axis with the number of particles gradually decreasing in the reaction flask suggest agglomerative growth or Ostwald ripening mechanisms at play.

Research on the nucleation and growth in colloidal semiconductor NCs has been mainly focused on binary NCs. It is implicitly assumed that the theories can be translated entirely for multielement colloidal nanocrystals. These real-time insights combining XAS and SAXS on the formation of quaternary CZTS nanorods reveals a stable intermediate exists in solution for a period of time after the anion injection and before any nucleation occurs. In addition, it is also revealed that early nuclei can be consumed to provide a source for the continuous growth of other nuclei. The results show that nucleation and growth in colloidal nanocrystals is more complex than that predicted by classical nucleation and growth theory and that identification of reaction specific intermediates and understanding of their temporal evolution is a key part of understanding their reaction pathways.

### Outlook

The herein applied combination of simultaneous XANES, EXAFS, and SAXS measurements reveals critical information about particle numbers, sizes, shapes, and the concentration of precursors, intermediates, and the final products in a colloidal nanocrystal synthesis. The ability to map to a specific element allows a complete picture to emerge of the nucleation and growth as composition evolves through binary, ternary to quaternary (in this case CZTS). The approach is generally applicable to any colloidal nanocrystal system and offers considerable opportunity to better understand key reaction intermediates in their formation. There are also opportunities with this simultaneous in situ monitoring to expand to additional measurements that can be simultaneously acquired (*e.g.*, optical spectroscopy). This would allow identification of minority phases with different optical properties that are not apparent below the XAS detection limit of ∼1%. In addition, further development of the data acquisition strategy toward measuring SAXS patterns for every energy point during an energy scan would allow the precision and reliability of the SAXS measurements to be further improved. The quantitative nature of this combination of techniques is also ideally suited to both inform and be informed by numerical modeling. The possibilities for this simultaneous in situ approach to enable a confluence of insights to emerge in the nucleation and growth processes across a wide range of colloidal nanocrystal chemistries is considerable.

## Conclusions

Combined in situ X-ray absorption spectroscopy and small-angle X-ray study of the colloidal synthesis of CZTS nanorods provides important insights into their growth mechanism. In particular, we have identified that a key copper thiolate intermediate forms instantaneously on thiol injection and decomposes further within a narrow temperature and time window to form copper sulfide NCs, which then transform to CZTS nanorods by the sequential incorporation of Sn and Zn into the lattice. The correlation with SAXS data in real-time shows that quasi-2D particles form initially, up to the point of bubbling, with secondary growth phase into rods having to occur by digestive growth as this elongation is occurring in the absence of Cu monomer. The findings of our work show that temporal occurrence of key reaction intermediates have a significant influence on reaction pathways in colloidal nanocrystal synthesis. It further shows that in situ monitoring of the progress from precursor through these intermediates to particle formation can allow for understanding of the kinetic pathways that enable their composition and shape evolution.

## Experimental Methods

### Materials

All reagents were used as received without any further purification. Copper(II) acetylacetonate (Cu(acac)_2_; > 99.99%), zinc acetate (Zn(Ac)_2_, > 99.99%), zinc(II) chloride (ZnCl_2_ > 99.99%), tin(IV) acetate (Sn(Ac)_4_, > 99.99%), trioctylphosphine oxide (TOPO, 99%), 1-dodecanethiol (1-DDT, 98%), tert-dodecylmercaptan (t-DDT, 98.5%, mixture of isomers), 1-octadecene (1-ODE, 90%, technical grade), and oleylamine (OLA, technical grade, 70%) were purchased from Aldrich.

### Synthesis of CZTS Nanorods

In a typical synthesis of CZTS nanorods, Cu(acac)_2_ (0.6545g, 2.5 mmol), Zn(Ac)_2_ (0.2292g, 1.25 mmol), Sn(Ac)_4_ (0.4435g, 1.25 mmol), TOPO (3.383g, 8.75 mmol), and 1-octadecene (25 mL) were added to a three-neck 50 mL round-bottom flask, fitted with reflux condenser, rubber septum, and temperature finger. (High precursor concentrations were used to ensure a high signal at the Cu K absorption edge.) The contents of the flask were evacuated at 100 °C for 30 min to eliminate adventitious water and dissolved oxygen. The reaction mixture was then heated to 230 °C (30 °C/min) under an argon atmosphere and at 155 °C, a 5 mL thiol mixture (composed of 4.375 mL t-DDT and 0.625 mL 1-DDT) was rapidly injected into the system. After injection, the reaction was allowed to proceed for 30 min with continuous stirring. Subsequently, the heating mantle was removed, and the reaction vessel was allowed to cool to 80 °C.

#### Washing Procedure

Initially, 2–3 mL of toluene was added to quench the reaction. The NCs were then washed twice with a 1:2 ratio of toluene to isopropanol (IPA) and centrifuged at 4000 rpm for 7 min. After each centrifugation, the supernatant was removed, and the precipitated nanorods were redispersed in toluene for further characterization.

### Reaction Flask and Heating Mantle

Specific modifications were made to the reaction flask to allow the X-ray beam to transmit through the flask for direct monitoring of NC nucleation and growth. A custom built three-neck round-bottom flask (50 mL capacity) was used for the reaction (Figure 1), which contained two 16 mm internal diameter inlet holes at opposing sides of the flask. Two stainless steel fittings were fixed into place at these inlets and were tightened to ensure no leakage between the glass flask and the fitting. Each fitting was designed in such a way as to contain a detachable segment at the top/innermost part, into which a “window” was placed to allow incoming and exiting X-ray beam to pass through the flask. This window was made from kapton foil, specifically cut to shape, and was positioned in the innermost part of the stainless steel fitting. The fitting was consequently tightened into place to prevent leakage. When both fittings were inserted into the flask, a 10 mm gap remained between the two fittings. During the reaction, the window and the area above these fittings was completely covered by the metal precursors and the solvent. Kapton was chosen for the window on the basis that it showed repeated ability to withstand the organic solvent—even at the elevated reaction temperatures—and also allows transmission of the X-ray beam through the flask.

### Anionic Precursor Injection System

A remotely controlled anionic precursor injection system was designed to capture the initial NC nucleation stage and was controlled from outside the beamline experimental hutch. The injection system comprised numerous components and valves, all of which worked in sequence to instigate a rapid and efficient injection at the desired injection temperature. The thiol mixture (anionic precursor) was filled into the injection tube, which was firmly sealed at the bottom by Kapton foil to prevent leakage. A piston was secured in the region above the thiol mixture in the tube. A combustion was ignited electronically, which pushed down the injection piston and injected the anionic precursor at a specific point in time.

### Quick-EXAFS Setup

Quick EXAFS data was measured at the SuperXAS Beamline at the Swiss Light Source in transmission mode using the QEXAFS monochromator, specially developed gridded ionization chambers and data acquisition schemes as described elsewhere in detail.^50−52^ The monochromator was set to oscillate with an amplitude of approximately 2.3° at a frequency of 20 Hz, resulting in the measurement of a complete spectrum from 8560 to 10075 eV every 25 ms, covering the XANES and EXAFS regions of the Cu– and Zn–K edges simultaneously. Transmission data and the encoder position of the Bragg angle were recorded with a frequency of 2 MHz.

### Quick-EXAFS Data Reduction

Data reduction and linear combination analysis of the as-recorded QEXAFS data were done with the JAQ software tool. The continuously recorded transmission data was binned in data points with an equal encoder value and split at the return points of the monochromator into individual spectra. Because of a slightly faster rise-time than fall-time of the used current amplifiers, only spectra with decreasing energy are taken into account for further evaluation. Energy calibration was performed on the final state of the reaction (CZTS NCs) according to externally measured reference spectra of stoichiometric CZTS powder (Cu K-edge: 8982.55 eV, Zn K-edge: 9661.8 eV).

## Supporting Information Available

Details on XANES, EXAFS, SAXS analysis, and TEM-EDS analysis on CZTS NCs (PDF)
