# Supplementary material for: Insights into Nucleation and Growth of Colloidal Quaternary Nanocrystals by Multimodal X-ray Analysis
Source: ACS Nano. 2021 Mar 26;15(4):6439–47. doi: 10.1021/acsnano.0c08617 (PMC8291568; doi:10.1021/acsnano.0c08617)
Supplement: Supplementary file 1 — nn0c08617_si_001.pdf [file nn0c08617_si_001.pdf]

Insights into Nucleation and Growth of Colloidal Quaternary Nanocrystals by Multimodal X-Ray Analysis

Justus Just,*[1] Claudia Coughlan,[2] Shalini Singh,*[2] Huan Ren,[2] Oliver Müller,[3] Pascal Becker,[4] Thomas Unold[4] and Kevin M. Ryan*[2]

1 MAX IV Laboratory, Lund University, Fotongatan 2, 22484 Lund, Sweden
2 Department of Chemical Sciences and Bernal Institute, University of Limerick, V94 T9PX Limerick Ireland
3 Stanford Synchrotron Radiation Lightsource, SLAC National Acceleration Laboratory, Menlo Park, California 94025, United States
4 Department of Structure and Dynamics of Energy Materials, Helmholtz-Zentrum Berlin für Materialien und Energie GmbH, 14109 Berlin, Germany

Content

Section S1 Details on XANES analysis

Section S2 TEM EDS analysis of CZTS nanocrystals

Section S3 Details on EXAFS analysis

Section S4 Details on SAXS analysis

Section S1 Details on XANES analysis:

XANES spectra are analyzed using the JAQ Software for QEXAFS data. Spectra are extracted, filtered in time, normalized and fitted with a linear combination of internal reference spectra.

Spectra are fitted with a linear combination of *internal* reference spectra (from the reaction itself). Which reference spectra from how many reference points during the reaction are taken is determined in an iterative process: First, the initial and final state of the reaction are taken as references only to fit all recorded spectra. In a second fit, an additional reference spectrum is taken at the position in time, where the deviation of the first fit from the measured data is at its maximum. These steps are iterated until the fit error -apart from noise- is constant over all measured spectra. While the LCA is performed on basis of individual spectra (50 ms) the reference spectra are taken from the respective reference point in time but averaged over 20 spectra (1 s) not to make the fit sensitive to individual noise on the reference spectra. To improve the clarity of the depicted LCA coefficients in Fig 2d and remove physically not relevant values (which arise *e.g.* from bubbling of the solution), a percentile filter over 50 points with a percentile of 50 was applied.

Cu K-edge: The energy was calibrated the final state XANES spectrum to 8982.55 eV as taken from an *ex situ* CZTS powder reference. The pre-edge line is fitted from -105.56 eV to -18.06 eV, while the post edge is fitted from 83.86 eV 323.75 eV referenced to the edge position. XANES reference spectra are produced taking an average of 20 spectra around the reference point in time. Linear combination fitting was performed in the spectral range from 8968.87 eV to 9074.57 eV.

Zn K-edge: The energy was calibrated the final state XANES spectrum to 9661.8 eV as taken from an *ex situ* CZTS powder reference. The pre-edge line is fitted from -134.58 eV to -28.16 eV, while the post edge is fitted from 158.17 eV to 413.95 eV referenced to the edge position. Reference spectra are produced taking an average of 20 spectra around the reference point in time.

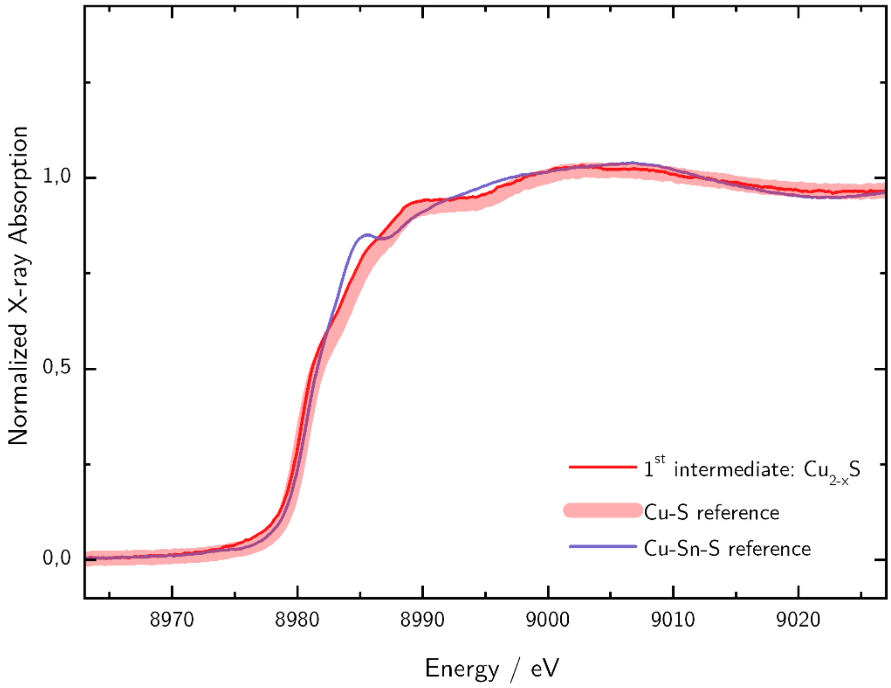

Figure S1.1. Edge step normalized X-ray absorption near edge structure (XANES) together with reference spectra recorded from the final sate of the same process with just Cu as well as Cu / Sn cation precursors.

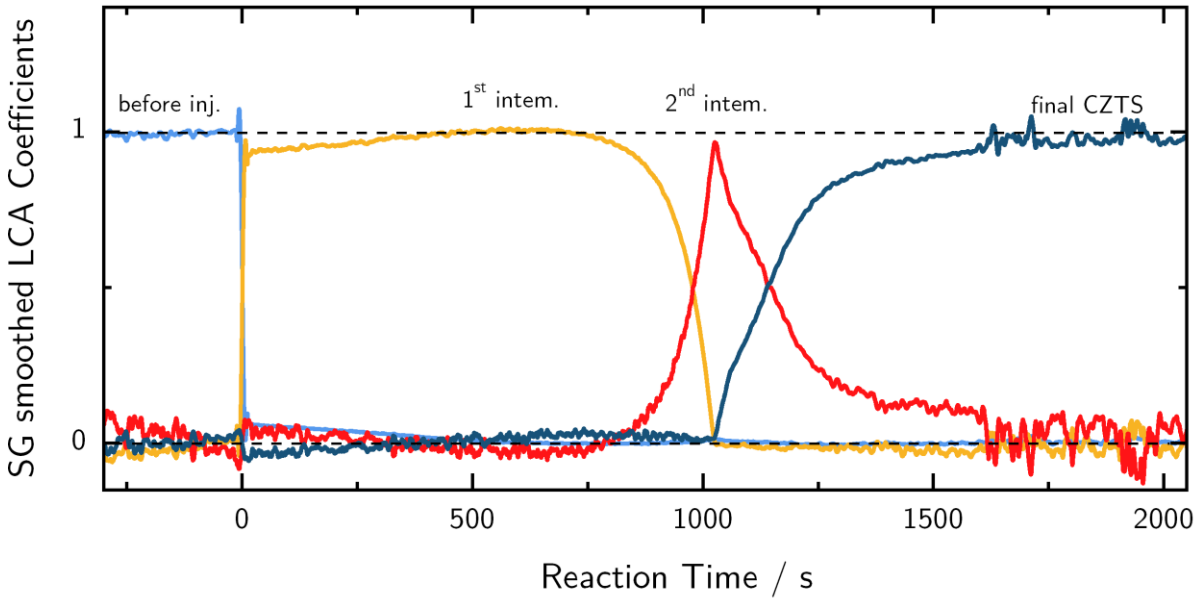

Figure. S1.2. Reaction kinetics as extracted from *in Situ* X-ray absorption spectroscopy at the K-edge of Copper by linear combination analysis using internal reference spectra from the indicated points of the reaction. Here the data from Figure 2d was smoothed applying a Savitzki-Golay filter over 500 points with a polynomial order of 4, as it was done on the raw spectra to extract EXAFS data. It is clearly visible that such a filter does not significantly smear out the kinetics.

Section S2 TEM EDS analysis of CZTS nanocrystals

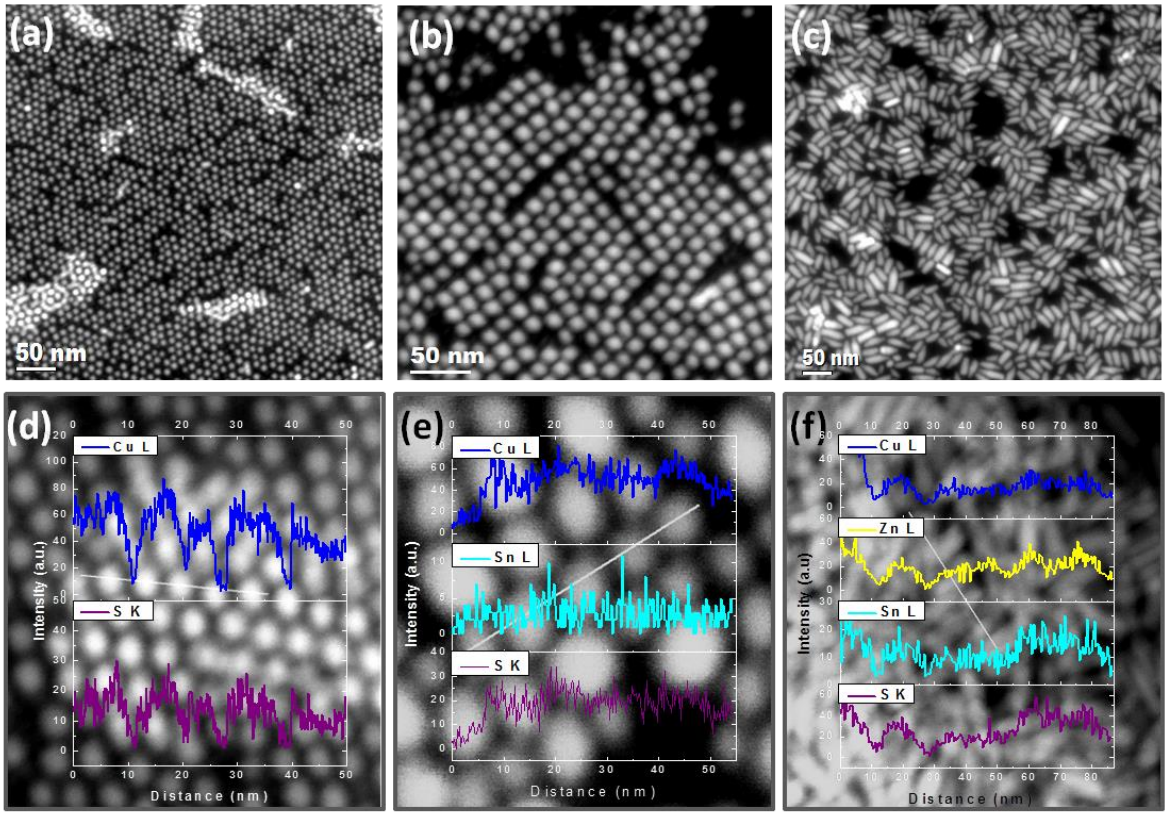

Figure S2.1. DF-STEM of aliquots taken at (a) 200°C, (b) 210°C and (c) 220°C. The corresponding higher magnification image and and elemental line scan analysis of aliquots taken at the aforementioned temperatures are shown in (d), (e) and (f) respectively.

Highly monodisperse spherical nanocrystals obtained at 200°C are shown in Figure S1. EDX line scan analysis of this sample across the measured line (depicted in Figure S1d) revealed that the nanocrystals were composed of two elements at this stage, specifically Cu and S. At 10°C above this aliquot, a slight morphological change was observed (Figure 5b), with EDX analysis of this sample detecting minor quantities of tin in the nanocrystals as shown in the line scan in Figure S1e. This signals the onset of tin incorporation into the nanocrystals at 210°C. At 220°C, bullet-shaped nanorods were formed in the reaction (Figure S1c) and EDX line scans across this sample detected equal quantities of both zinc and tin in the nanorods (Figure S1f). These observations clearly identify the region between 200°C and 220°C as crucial in the formation of CZTS nanorods, with both tin and zinc incorporating into the pre-formed $Cu_{2-x}S$ nuclei, respectively, and within a short timeframe in this narrow temperature range.

Section S3 Details on EXAFS analysis

EXAFS spectra are generated by applying a 4th order Savitzky-Golay filter on 500 spectra around the respective reference point in time. EXAFS spectra are extracted as described in the article and are analyzed using Athena, Atremis and FEFF from the Demeter software package.

1) **Cu K-edge: k-range**: 2.856-12.119 /A

Cu-precursor: quick 1st shell theory with Oxygen shell

k-range: 1.5-12.119/A; Fit in R-space: 1-1.845A (not phase corrected) k-weight: 3

Fit results: aa: see graphics@N=1; e0: 3.9 eV; dr: see graphics; MSRD: 0.0042+-0.0008A²

1st intermediate: quick 1st shell theory with Sulfur shell

Fit in R-space: 1-2.186A (not phase corrected) k-weight: 2,3

Fit results: aa: see graphics@N=1; e0: 7.0 eV; dr: see graphics; MSRD: 0.0053+-0.0007A²

2nd intermediate: quick 1st shell theory with Sulfur shell

Fit in R-space: 1-2.8A (not phase corrected) k-weight: 2,3

Fit results: aa: 2.0 ± 0.2@N=1; e0: 5.7 eV; dr: see graphics; MSRD: 0.010+-0.002A²

Final state CZTS: 1st coordination shell: Sulfur in wurtzite structure generated from wurtzite ZnS with replacement of Zn partially by Cu and Sn according to the stoichiometry.

Fit in R-space: 1-2.6A (not phase corrected) k-weight: 3

Fit results: aa: 0.91 +- 0.04, d: see graphic, e0: 1.2eV, N=4; Einstein model for MSRD with values determined from powder reference, see EXAFS paper: Teinst: 309 K, T=485 K

2) Zn K-edge: k-range: 2-8.405 /A

Zn-precursor: quick 1st shell theory with Oxygen shell

k-range: 2-8.405/A; Fit in R-space: 0.9-2.144A (not phase corrected) k-weight: 2,3

Fit results: aa: 3.6+-0.16@N=1; e0: 0.84+-0.4 eV; R: 1.975+-0.004A; MSRD: 0.0078+-0.0008A²

Zn-after injection: quick 1st shell theory with Oxygen shell

k-range: 2-8.405/A; Fit in R-space: 0.9-2.144A (not phase corrected) k-weight: 2,3

Fit results: aa: 3.6+-0.2@N=1; e0: 1.73+-0.6 eV; R: 1.971+-0.006A; MSRD: 0.0075+-0.001A²

Zn-final: quick 1st shell theory with Sulfur shell

k-range: 2-8.405/A; Fit in R-space: 1-2.927A (not phase corrected) k-weight: 2,3

Fit results: aa: 3.5+-0.5@N=1; e0: 5.0+-1.3eV; R: 2.363+-0.06A; MSRD: 0.0074+-0.002A²

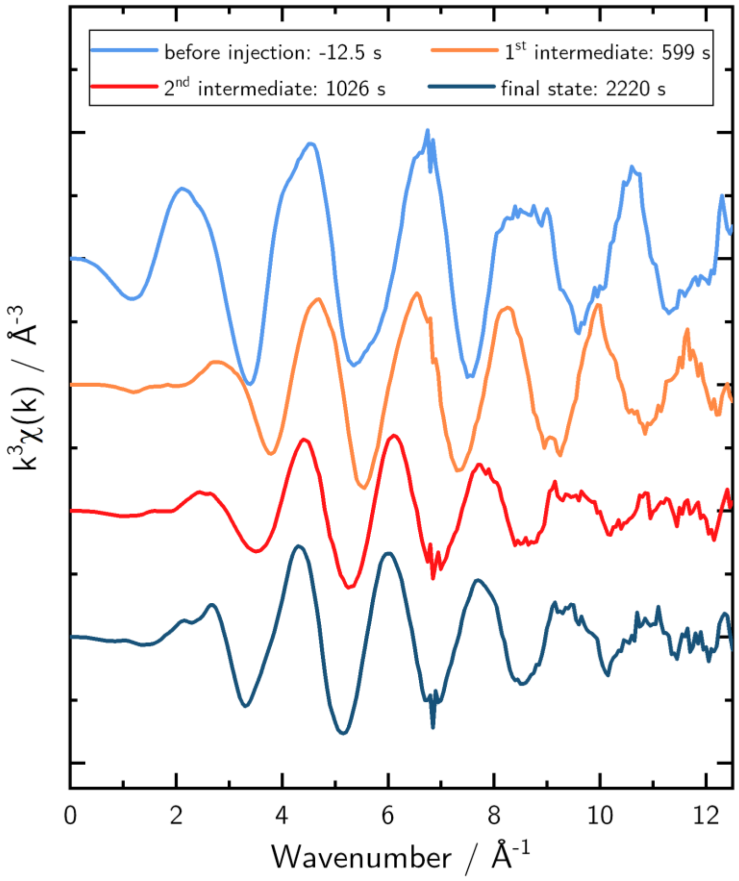

Figure S3.1. Extracted and k3-weighted EXAFS oscillations of the k-edge of Cu measured after different times of the ongoing reaction. This data is basis for Fig. 3b.

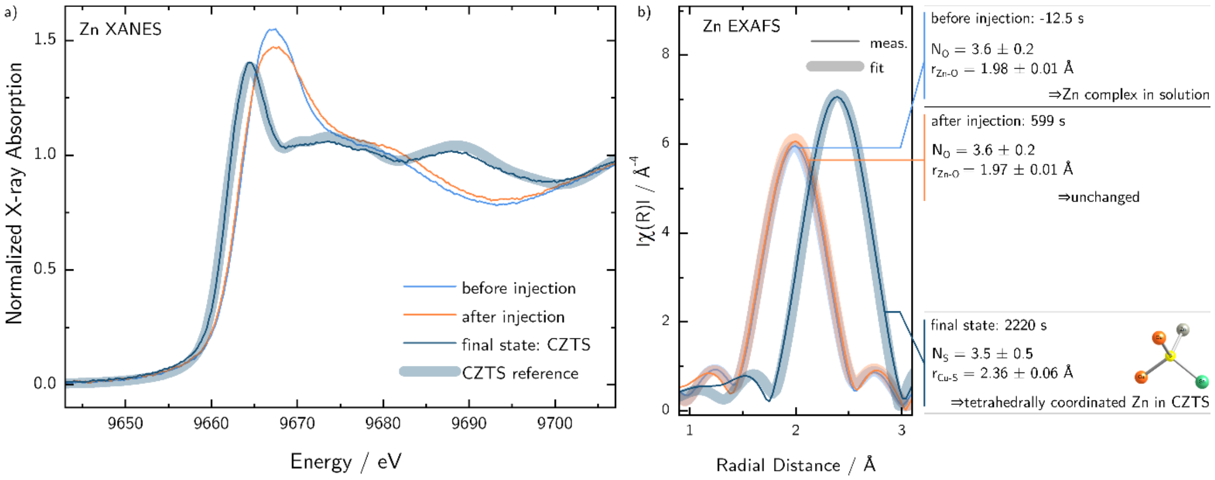

Figure S3.2. Extracted Zn K-edge X-ray absorption spectra measured *in Situ* at different times of the reaction. a) Edge step normalized X-ray absorption near edge structure (XANES) together with powder reference spectra of stoichiometric tetragonal kesterite CZTS. b) Phase corrected Fourier transform of the EXAFS oscillations measured at different times of the reaction together with a fitted *ab initio* model to extract coordination numbers and bond lengths. The as determined local surrounding of Zinc is shown schematically on the right.

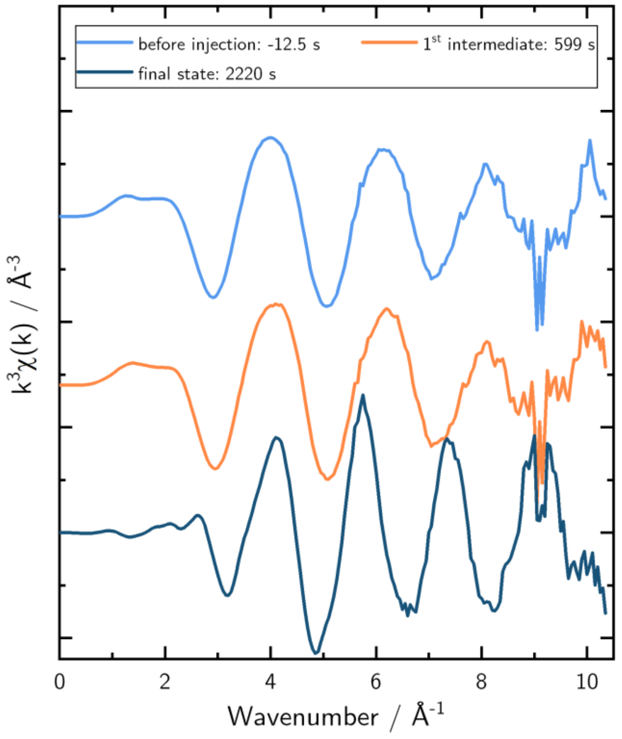

Figure S3.3. Extracted and k3-weighted EXAFS oscillations of the k-edge of Zn measured after different times of the ongoing reaction. This data is basis for Figure S3.2b.

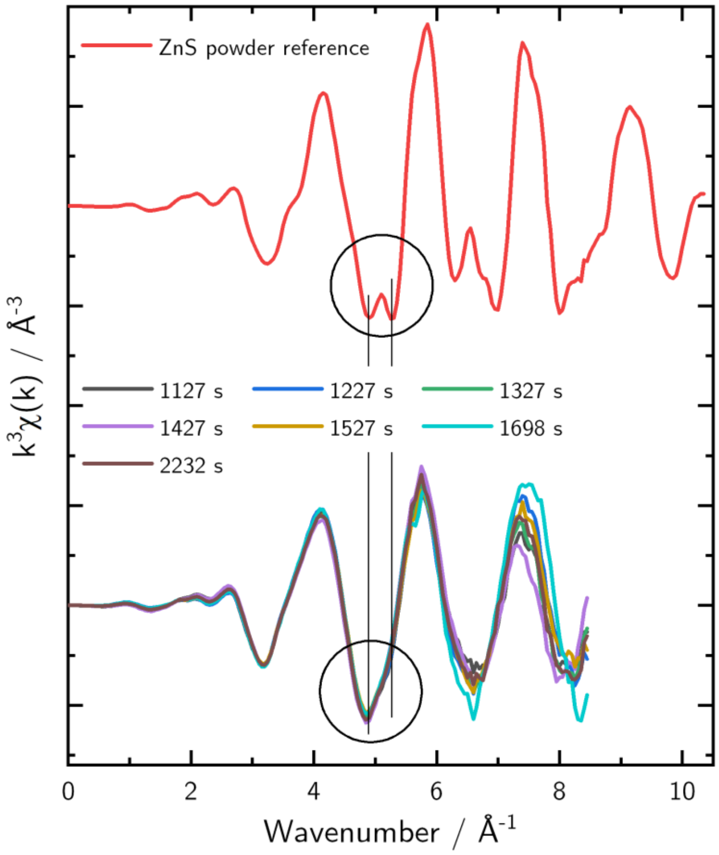

Figure S3.4. Extracted and k3-weighted EXAFS oscillations of the k-edge of Zn measured after different times of the ongoing reaction, as indicated by red crosses in Fig. 4c, as well as from ZnS reference powder.

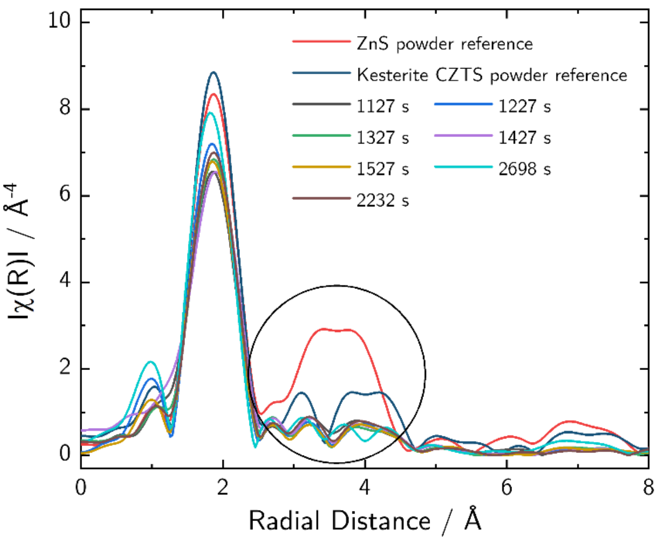

Figure S3.5. Fourier transform (not phase corrected) of the extracted and k3-weighted EXAFS oscillations of the k-edge of Zn measured after different times of the ongoing reaction, as indicated by red crosses in Fig. 4c, as well as from ZnS and Kesterite CZTS reference powders. The lower contribution to the first metal shell, here around 3.5 A, of the wurtzite nanocrystals compared to kesterite CZTS can be attributed to the increased cation disorder in case of the wurtzite structure.

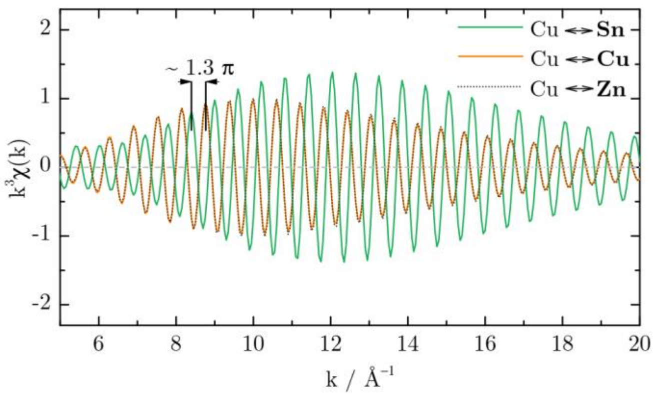

Figure S3.6. Calculated EXAFS oscillations of single scattering paths originating from a copper atom and backscattered at Sn, Cu and Zn based on the *ab initio* calculated backscattering amplitudes and phases from Figure 8 with an effective path length of r = 5.4 A and a MSRD σ2 = 0.003 A

Section S4 Details on SAXS analysis

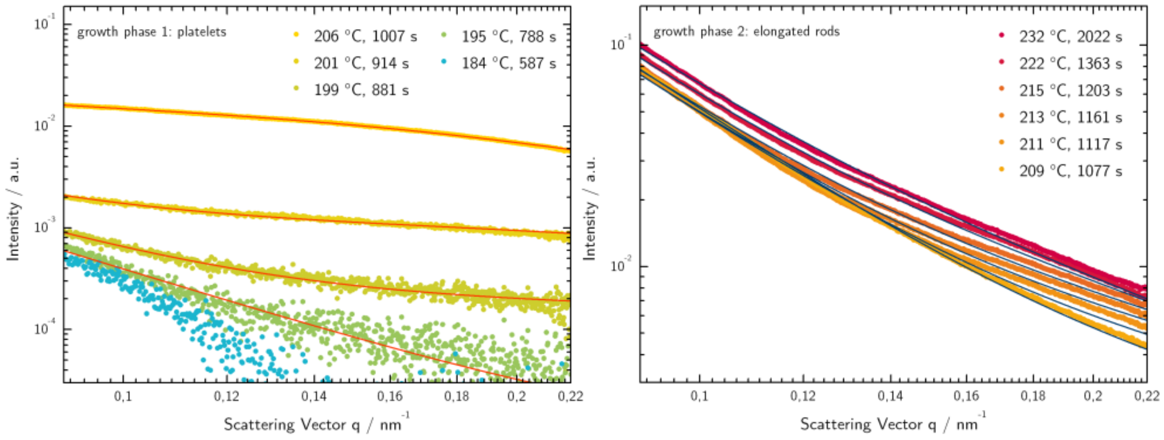

Figure S4.1. *In situ* measured SAXS scattering patterns extracted from radial integration of the scattering images for different temperatures / times of the reaction. For clarity, patterns are shown for the different stages of growth or before and after the bubbling in the reaction flask in separate figures with different scaling. Fits using a Porod background (exponent -4) together with a cylinder model are shown as red and blue lines, respectively.

The SAXS patterns after bubbling in the reaction flask (Figure S3 right) show significant agglomeration resulting in a strong Porod-like background. Ignoring this, the total volume fraction of particles in solution –as obtained by fitting the SAXS patterns- does not follow the expected behavior from the XANES signals LCA fits.

In order to still extract valuable information, the patterns are fitted with altered models before and after this agglomeration occurs, as described in the following.

- All patterns are fitted between 0.09 and 0.22 1/nm
- The SASview software package was used to fit the individual patterns

Before bubbling of the solution: The point, where 100% of the Cu is in solid $Cu_{2-x}S$ (from XANES) was taken as a reference point to start the fitting. SAXS patterns therefore can be treated in a quantitative way. The volume fraction was calculated based on the total concentration of Cu-precursor salt reacted fully into $Cu_{2-x}S$ particles resulting in 0.00113. The SAXS pattern was fitted with a cylinder + Porod (exp-4) P(Q) model together with the assumption of hard spheres for S(Q). The only free variables were the total scale, the Porod scaling and the radius of the cylinders. The length was fixed to 1nm because of the assumption of platelets (as seen from aliquots). The exact value is arbitrary and does not significantly influence the fit in this region. The only thing, which is influenced is the absolute scaling and the exact value of the radius.

All following patterns until the bubbling in the reaction flask (and agglomeration) begins are fitted with the same model, keeping the scaling constant as determined, while varying only the radius and Porod scale. The volume fraction for each point was fixed to the value obtained from XANES.

Polydispersity is needed in order to describe the SAXS patterns well, see Figure S4. The exact value of the polydispersity influences the resulting value of the radius but was arbitrarily fixed to 1. Therefore, the radius cannot be taken as an absolute measure.

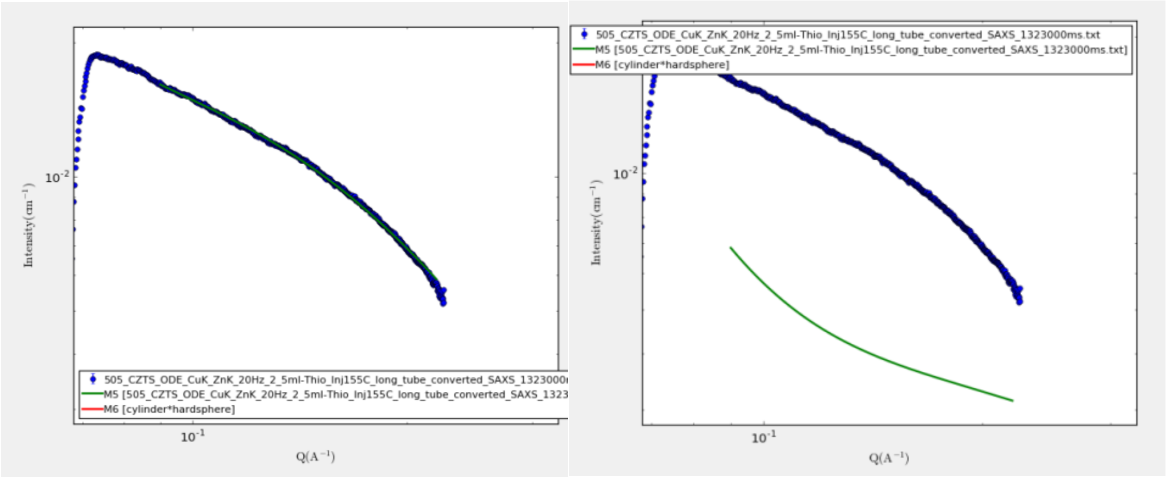

Figure S4.2. Fits of the reference point with and without polydispersity = 1r (Gaussian)

For radii smaller than 1nm the length was set to the value of the double radius to come closer to the symmetrical points. Due to the background level, it was not possible to fit for times < 788s.

After bubbling in the solution:

all patterns after the agglomeration show a strong prod contribution (from agglomeration) and a much weaker signal of the particles itself. As reference pattern, a pattern from the end of the reaction is taken.

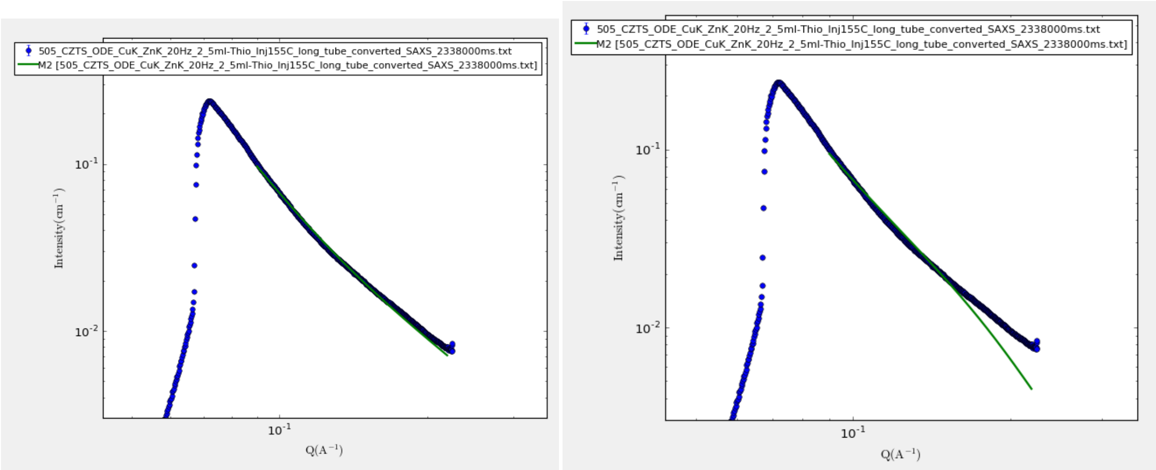

Figure S4.3. Fits of the final reference pattern without and with polydispersity = 1r

Polydispersity was therefore disabled for all fits after the bubbling in the reaction flask. The pattern was fitted with the porod + cylinder model fixing the radius and the scaling to the values from before the bubbling in the reaction flask. The final length was fixed to 30 nm according to a comparison with TEM images. The polysidpersity in lengths was estimated from TEM images to be around 30%, which has no significant effect on the SAXS pattern. The only free variables during the fit were the volume fraction of particles in solution and the porod scale. After evaluating the final pattern, all patterns after the bubbling in the reaction flask were fitted with this model, keeping every value as determined from the final pattern and varying the length and the Porod scale only.

The volume fraction of “free” (not agglomerated) particles was determined to 0,00018 which is about 10% of this before agglomeration.

Further processing:

Because of the specific implementation of the porod + cylinder model in SASview the porod scale is relative and must be corrected /radius^2/length to obtain the porod contribution to the pattern. The porod contribution can be used as a measure of agglomeration.

Further the particle density - the number of particles per volume are calculated based on the volume fraction together with the single particle volume as determined from the fit. After the bubbling in the reaction flask, the absolute volume fraction was corrected by adding the single particle SAXS volume fraction together with a proportionality factor times the porod contribution to estimate the number of particles as it would be without agglomeration. The proportionality factor was determined taking the final reference pattern as a measure to reach the final number of particles as it can be calculated from the particle dimensions together with the weighted precursor amounts.
